# Endoscopic Retrograde Cholangiopancreatography (ERCP)-Related Duodenal Perforation: Management Challenges and Lessons From a Case Series

**DOI:** 10.7759/cureus.89398

**Published:** 2025-08-05

**Authors:** Duc N Nguyen, Lai T Vo, Hung V Vo, Dung T Ho

**Affiliations:** 1 Liver Cancer Department, Binh Dan Hospital, Ho Chi Minh, VNM

**Keywords:** aggressive drainage, duodenal perforation, ercp, jejunal feeding, stapfer classification

## Abstract

Duodenal perforation is a rare but harmful complication of endoscopic retrograde cholangiopancreatography (ERCP). Early diagnosis and appropriate management are critical to reduce morbidity and mortality. Four patients, aged 36 to 56 years, underwent ERCP for biliary obstruction due to choledocholithiasis or postoperative biliary stricture. Symptom onset ranged from 12 to 40 hours post-ERCP. Imaging revealed retroperitoneal air, periduodenal fluid collections, or free intraperitoneal air. Three patients were initially managed conservatively. Two of them subsequently required delayed surgical intervention due to clinical deterioration. One patient was treated non-operatively throughout the hospital stay but did not recover; the family declined surgery. Another patient underwent early operative management based on overt signs of generalized peritonitis and pneumoperitoneum. Duodenal perforation was classified as Stapfer type II in three cases and type I in one case. This case series highlights the clinical variability and diagnostic challenges associated with ERCP-related duodenal perforations. Accurate classification using the Stapfer system and timely diagnosis with contrast-enhanced CT are crucial in guiding management. While conservative treatment may be effective in selected patients, delayed recognition or clinical deterioration often necessitates surgical intervention. Concomitant complications, such as acute pancreatitis, necrotizing cholecystitis, liver cirrhosis, or ampullary bleeding, can affect the clinical picture of perforation and worsen the overall prognosis. Aggressive drainage and enteral feeding via jejunostomy contributed positively to local control of pancreatic inflammation and promoted duodenal healing. Prompt diagnosis and multidisciplinary approach strategies are key to optimizing outcomes in ERCP-related duodenal perforations.

## Introduction

Endoscopic retrograde cholangiopancreatography (ERCP) is a commonly used procedure for diagnosing and treating biliary diseases. Despite its benefits, ERCP is associated with complications, including pancreatitis, bleeding, and perforation. Duodenal perforation, although rare, remains one of the most severe and potentially fatal complications, with an estimated incidence of 0.1-1.5% and a high risk of morbidity and mortality if not promptly identified and managed [1]. Perforations can occur as a result of mechanical trauma from the endoscope, sphincterotomy, or instrumentation such as guidewires or baskets. The Stapfer classification categorizes these injuries into four types based on the location and mechanism of injury, supporting clinical decisions [2]. A recent systematic review revisited over five decades of data on ERCP-induced perforation, emphasizing the importance of Stapfer classification in guiding management strategies and predicting clinical outcomes [3]. However, the symptoms of duodenal perforation are often nonspecific and can overlap with post-ERCP pancreatitis [4]. Contrast-enhanced computed tomography (CT) is critical for diagnosis, but therapeutic strategies must be individualized based on the patient's stability, type of perforation, and concomitant complications [3,5].

In this report, we present a series of four patients with ERCP-related duodenal perforation managed with varying strategies, including conservative therapy and surgery. We aim to highlight the diagnostic challenges, decision-making process, and clinical outcomes, while reviewing current management principles for this complex complication.

## Case presentation

Case 1

A 36-year-old male with a history of type 2 diabetes mellitus and liver cirrhosis was admitted for the management of choledocholithiasis. ERCP was performed, revealing a mildly dilated common bile duct (CBD; 5 mm) with biliary sludge. There was notable fibrotic narrowing of the sphincter of Oddi, making guidewire and sphincterotomy challenging. A wide sphincterotomy was performed, and sludge was successfully removed. A nasobiliary drain was then placed in the left and right hepatic ducts under C-arm fluoroscopy.

**Figure 1:**
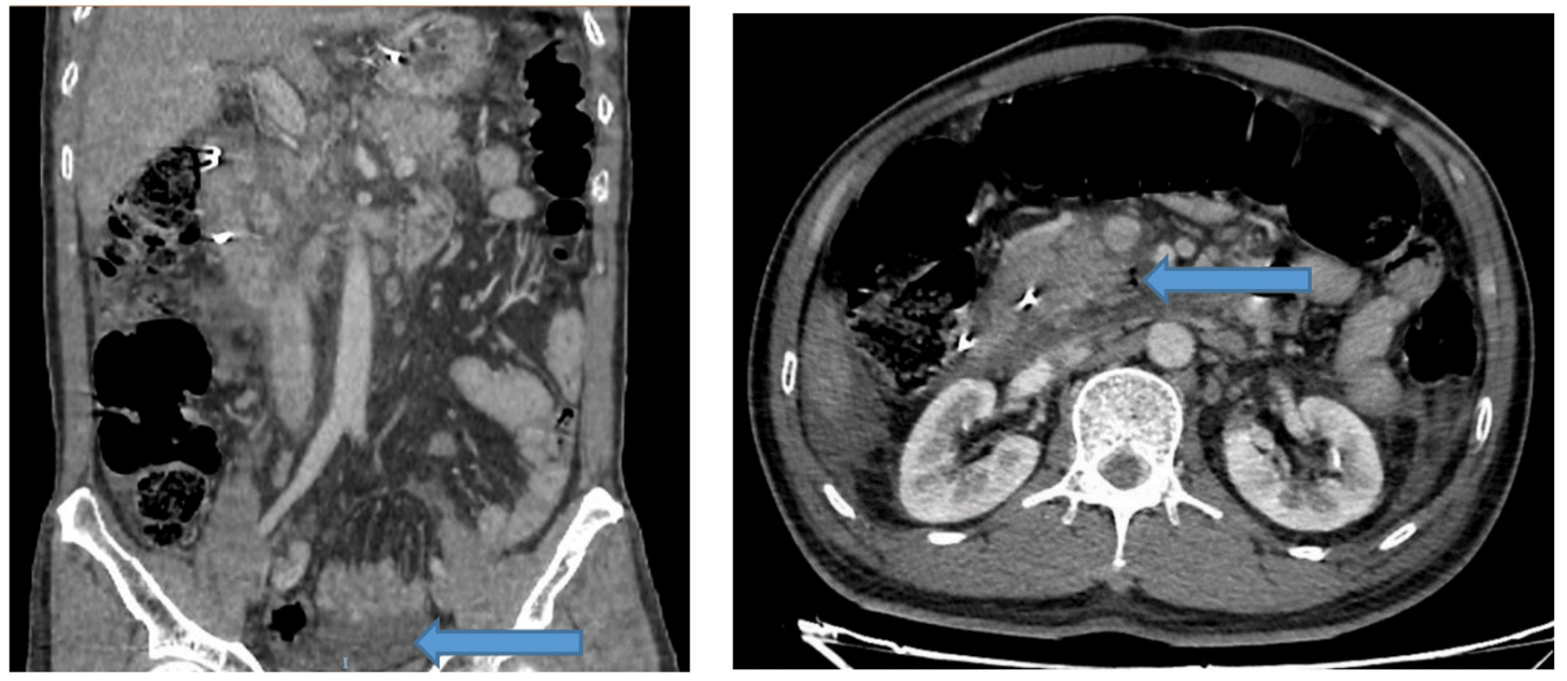
Computed tomography showed retroperitoneal free air in the peripancreatic and periduodenal regions and moderate ascites and mesenteric fat stranding.

Approximately 12 hours post-ERCP, the patient developed epigastric pain and increasing abdominal distension. Laboratory evaluation revealed leukocytosis (WBC 22,000/μL; reference range: 4,000-11,000/μL) and elevated serum amylase (2097 U/L; reference range: 30-110 U/L). A contrast-enhanced abdominal CT scan demonstrated retroperitoneal free air in the peripancreatic and periduodenal regions, moderate ascites, and mesenteric fat stranding (Figure 1). Additionally, imaging findings were consistent with edematous pancreatitis (CT severity index (CTSI) = 4). Since the patient's stable hemodynamic status, conservative management was pursued by bowel rest, nasogastric decompression, and broad-spectrum antibiotics.

**Figure 2:**
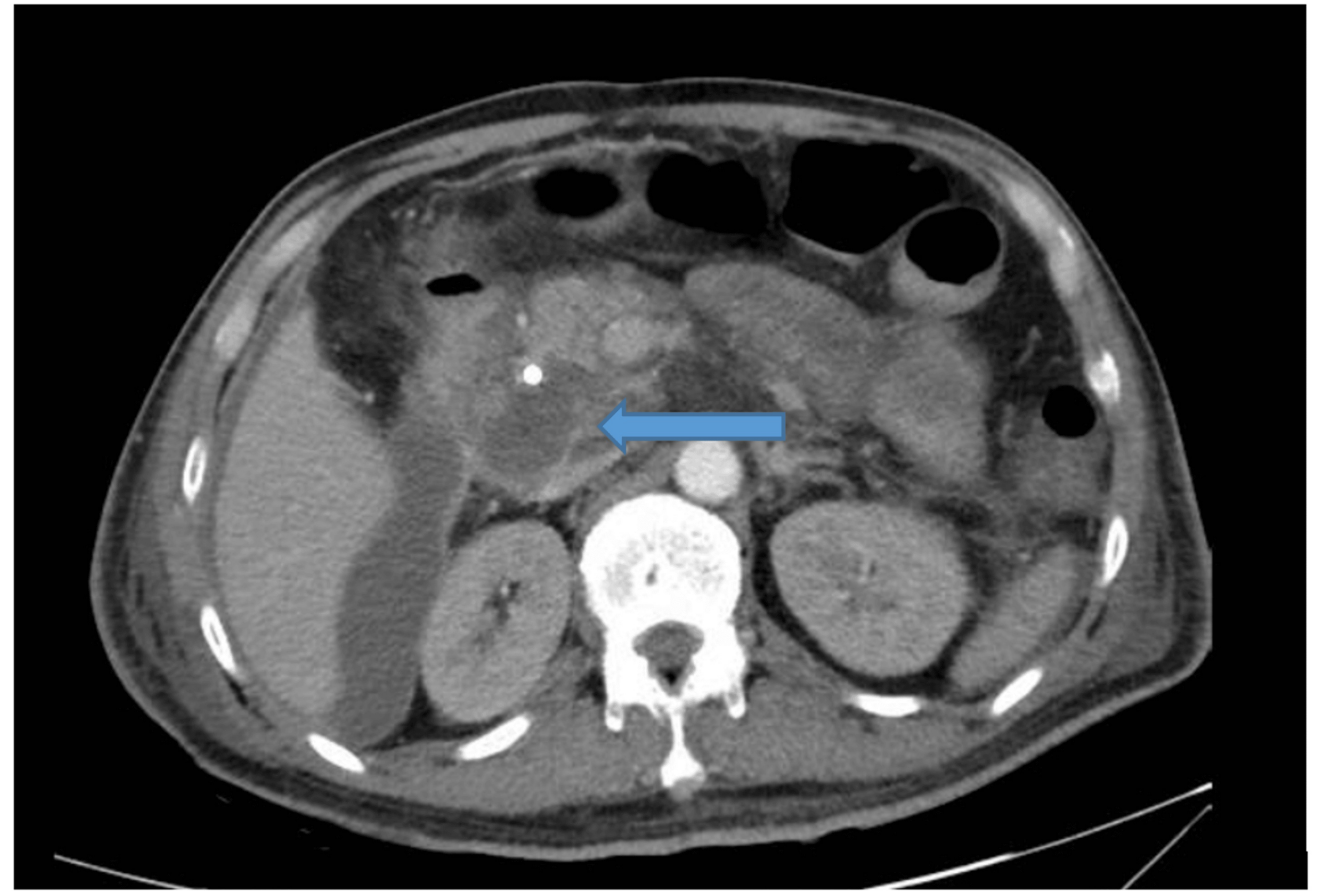
CT scan showed a persistent fluid collection (13 × 30 mm) posterior to the second and third portions of the duodenum.

On post-procedure day 9, the patient experienced hematemesis. Urgent upper gastrointestinal endoscopy revealed active bleeding from the ampulla of Vater, with adherent clotted blood on the duodenal wall. Hemostasis was achieved using epinephrine injection. A repeat CT scan demonstrated a walled-off retroperitoneal perforation and a persistent fluid collection (13 × 30 mm) posterior to the second and third portions of the duodenum (Figure 2).

Despite intensive supportive care in the ICU, including hemodynamic stabilization and infection control, the patient showed minimal clinical improvement over a 29-day hospital stay. He remained intubated and mechanically ventilated, with persistent hypotension requiring vasopressor support (norepinephrine and adrenaline). Ongoing signs of sepsis, decompensated cirrhosis with ascites, and bleeding from the ampulla of Vater were noted. The duodenal perforation was classified as Stapfer type II. His family ultimately decided to discharge him against medical advice. The unfavorable outcome was likely driven by the combination of septic shock, cirrhosis, viral hepatitis B, and post-ERCP complications.

Case 2

A 36-year-old female with a history of laparoscopic cholecystectomy presented with recurrent right upper quadrant pain. ERCP was indicated due to suspected biliary obstruction. Cholangiography revealed a mildly dilated CBD (10 mm) with a near-complete distal CBD stricture that prevented advancement of the guidewire and cutting device beyond the stricture, despite repeated attempts. Endoscopic sphincterotomy (ES) was done. The endoscopy team recommended early surgical decompression because of the inability to relieve the obstruction.

**Figure 3:**
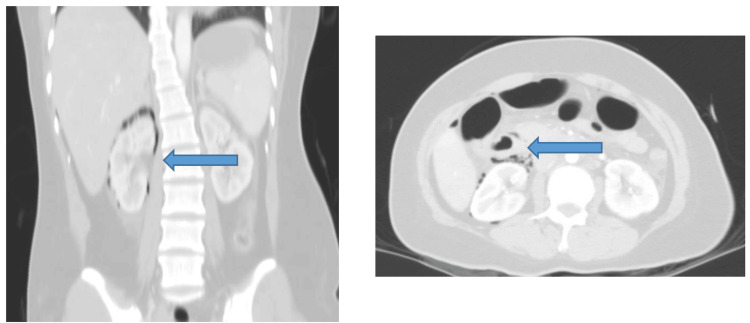
CT showed large volumes of free intraperitoneal air, predominantly around the pancreatic head and duodenum.

Approximately 13 hours post-ERCP, the patient developed acute epigastric pain radiating to the back. Laboratory tests revealed leukocytosis (WBC 13,000/μL; reference range: 4,000-11,000/μL) and elevated serum amylase (2,271 U/L; reference range: 30-110 U/L). A contrast-enhanced CT scan demonstrated large volumes of free intraperitoneal air, predominantly around the pancreatic head and duodenum, with limited retroperitoneal air extending to the right perirenal space, along with mild peripancreatic fat stranding (Figure 3). These findings were most consistent with an anterior duodenal wall perforation and classified as Stapfer Type I.

The patient underwent urgent exploratory laparotomy. The Kocher maneuver and duodenal mobilization were performed. No obvious perforation site or methylene blue leakage was identified. The CBD was incised, purulent bile was evacuated, and a single distal CBD stone was removed. A T-tube was inserted for biliary drainage. Surgical drains were placed appropriately.

The postoperative period was remarkably smooth, with no requirement for intensive care support. The patient showed rapid recovery, with stable vital signs and clinical improvement. Early mobilization and effective nutritional support contributed to her swift recovery. She was discharged after just 14 days of hospitalization, marking the fastest recovery among the four cases.

Case 3

A 56-year-old male with a history of biliary surgery (operative records unavailable) presented with clinical features of acute cholangitis. Laboratory evaluation showed leukocytosis (WBC 18,220/μL; reference range: 4,000-11,000/μL). Emergency ERCP revealed multiple distal stones (5-10 mm) and dense biliary sludge in the CBD. Significant narrowing of the common hepatic duct (3-4 mm) was also noted, preventing access to the left hepatic duct for complete stone removal. Endoscopic sphincterotomy was performed, followed by stone removal using a Dormia basket. Despite thorough flushing, residual stones in the left hepatic duct could not be accessed due to the tight stricture. To achieve decompression, a nasobiliary drain was placed into the left hepatic duct, and plans were made for a second ERCP to complete stone clearance.

**Figure 4:**
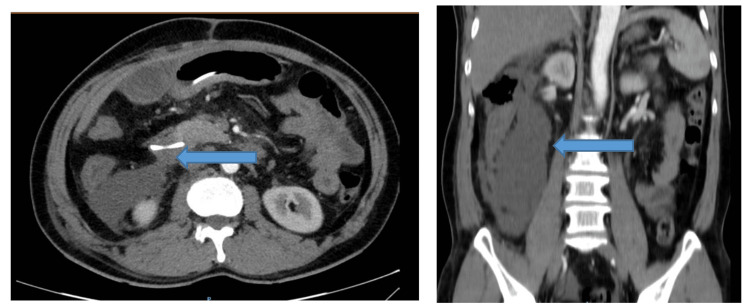
CT demonstrated a loss of continuity of the posterior duodenal wall at the second portion (D2), with a retroperitoneal fluid and gas collection extending to the right perirenal and paracolic spaces.

Approximately 40 hours post-ERCP, the patient developed severe epigastric pain. While he remained hemodynamically stable, physical examination revealed mild abdominal distension and tenderness. Contrast-enhanced CT demonstrated a loss of continuity of the posterior duodenal wall at the second portion (D2), with a retroperitoneal fluid and gas collection extending to the right perirenal and paracolic spaces -- findings consistent with retroperitoneal duodenal perforation (Stapfer type II). Additionally, the CT revealed inflammatory changes around the gallbladder, loss of wall integrity, and pericholecystic fluid, suggesting necrotizing cholecystitis (Figure 4).

**Figure 5:**
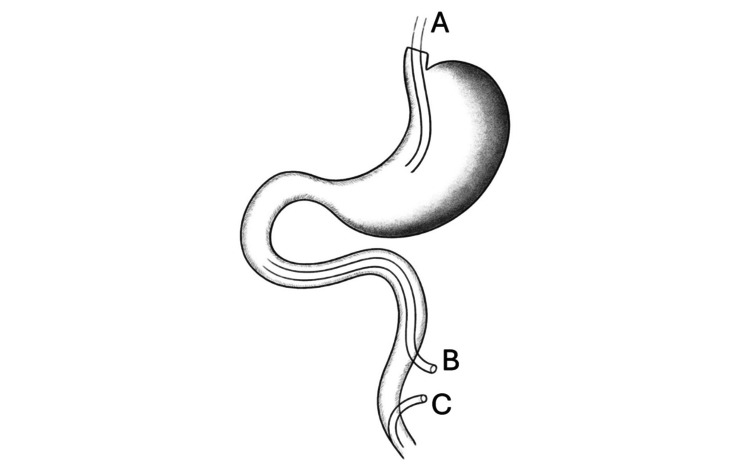
Diagram of the intraoperative two-tube technique A: Nasogastric tube, B: Decompression tube, C: Feeding jejunostomy tube This figure was created by the authors for illustrative purposes and is not reproduced from any published source.

Considering the diagnosis of necrotizing cholecystitis and the risk of sepsis, the patient was taken for urgent exploratory laparotomy. The gallbladder was necrotic and was removed. A Kocher maneuver and retroperitoneal dissection were performed, revealing a localized inflammatory cavity posterior to the duodenum. No active methylene blue leakage was identified. The CBD was incised, the purulent bile and residual stones were removed, and the left hepatic duct was dilated. A T-tube was placed for external biliary drainage. Two Pezzer tubes were inserted via the jejunum: one directed proximally for duodenal decompression, and the other used as a feeding jejunostomy to maintain enteral nutrition (Figure 5). Appropriate surgical drains were placed.

The postoperative course was uneventful, and the patient was discharged in stable condition on postoperative day 32.

Case 4

A 48-year-old female with a history of laparoscopic cholecystectomy performed in 2023 was admitted for evaluation of biliary obstruction. ERCP demonstrated a markedly dilated proximal CBD (15 mm), a short fibrotic stricture in the mid-segment measuring 3 mm in length with a 2 mm diameter, and a large impacted stone (15 mm). Endoscopic sphincterotomy was performed with an attempt at stone removal through a Dormia basket. Due to the size of the stone, lithotripsy was required to fragment it into smaller pieces. Several fragments were successfully removed; however, residual stones remained within the tight stricture. A nasobiliary drain was placed, and its position was already confirmed via C-arm fluoroscopy as it was seen extending into the left hepatic duct. Plans were made for a second ERCP to complete stone clearance.

**Figure 6:**
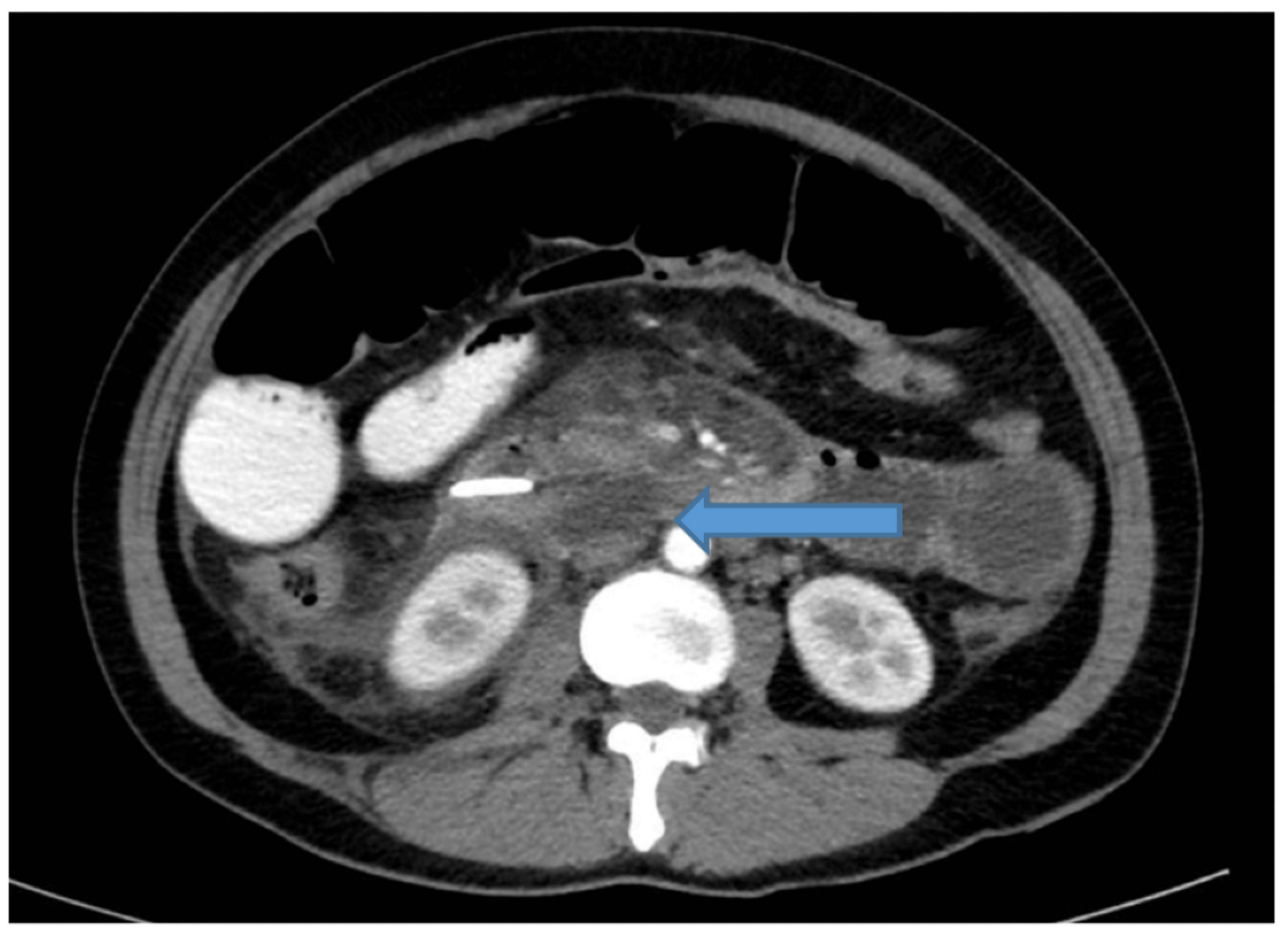
CT scan showed a 15 × 37 mm retroperitoneal fluid and gas collection posterior to the D2-D3 segments of the duodenum.

At 21 hours post-ERCP, the patient reported severe epigastric pain. She remained hemodynamically stable initially, with elevated serum amylase (2,576 U/L; reference range: 30-110 U/L). On post-procedure day 3, she developed progressive abdominal distension and systemic signs of deterioration. By day 4, she presented with tachycardia, livedo reticularis, hypoxemia (oxygen saturation (SpO₂) 91%), and worsening abdominal distension, prompting transfer to the intensive care unit. Contrast-enhanced CT scan revealed a 15 × 37 mm retroperitoneal fluid and gas collection posterior to the D2-D3 segments of the duodenum, consistent with a Stapfer type II duodenal perforation, along with edematous pancreatitis (CTSI = 3) (Figure 6).

Due to rapid clinical deterioration and persistent retroperitoneal abscess formation, urgent exploratory laparotomy was performed. A Kocher maneuver revealed extensive inflammation and necrosis involving the duodenum and pancreatic head, with pseudomembrane formation suggestive of evolving necrotizing pancreatitis. Although no overt duodenal perforation was visualized, a purulent retroperitoneal collection was drained. The CBD was incised, and residual stone fragments were removed. A T-tube was placed for external biliary drainage. A feeding jejunostomy was made using a Pezzer tube to maintain enteral nutrition, allowing adequate time for duodenal healing. Additionally, two drainage catheters were strategically placed around the pancreatic bed: one for irrigation and the other for drainage, establishing a continuous lavage system aimed at controlling local inflammation and necrosis.

Postoperatively, the patient required mechanical ventilation, continuous renal replacement therapy (CRRT), and transfusions for septic shock and multiorgan failure. So, a second-look laparotomy was performed on postoperative day 5 for drain revision and replacement of the feeding tube. Despite initial signs of leakage, effective drainage from the peripancreatic and retroperitoneal spaces combined with sump irrigation facilitated infection control and promoted healing of the duodenal perforation. Nutritional support via jejunal feeding contributed to recovery and stabilization. The patient gradually improved, and although she was discharged with a peripancreatic drain in place after a 51-day hospitalization, her condition was stable and well-managed.

A comparative summary of the clinical characteristics, diagnostic findings, and management approaches for all four patients is provided in Table 1.

**Table 1:** Clinical characteristics and management of four patients with ERCP-related duodenal perforation CBD: common bile duct, CHD: common hepatic duct, ERCP: endoscopic retrograde cholangiopancreatography

Parameter	Case 1	Case 2	Case 3	Case 4
Age/Gender	36/Male	36/Female	56/Male	48/Female
ERCP Findings	CBD sludge, Oddi stricture	CBD multiple stones and sludge, CHD stricture	CBD multiple stones, Left hepatic duct stricture	CBD large stone, Mid-CBD stricture
Interventions during ERCP	Sphincterotomy, Sludge removal, Nasobiliary drainage	Sphincterotomy attempted, incomplete due to a tight stricture; Recommended early surgical decompression	Sphincterotomy, Part of the stone removal, Nasobiliary drainage, Another ERCP was planned for complete stone removal	Sphincterotomy, Lithotripsy, Nasobiliary drainage
Time to Symptom Onset	12 hours	13 hours	40 hours	21 hours
Imaging Findings	Free air around the pancreas and duodenum, Pancreatitis edema (CTSI 4)	Large free air around the head, pancreas, and duodenum	Retroperitoneal air – loss of continuity of the wall of the duodenum, Necrotizing cholecystitis	Retroperitoneal fluid and gas, Pancreatitis edema (CTSI 3)
Stapfer Classification	Type II	Type I	Type II	Type II
Surgical Indication	Not operated – surgery declined by the family	Duodenum perforation	Necrotizing cholecystitis	Failed conservative management
Management	Conservative management	Surgical, Stone removal, Biliary drainage	Surgical, Cholecystectomy, Stone removal, Biliary drainage, Feeding jejunostomy	Surgical, Stone removal, Biliary drainage, Feeding jejunostomy
ICU Requirement	Yes	No	Yes	Yes
Length of Hospital Stay	29 days	14 days	32 days	51 days
Outcome	Discharged against medical advice (AMA)	Full recovery, discharged stable	Full recovery, discharged stable	Discharged with drains, stable

## Discussion

Endoscopic retrograde cholangiopancreatography is an essential therapeutic procedure for biliary and pancreatic disorders; however, it carries a risk of complications, including duodenal perforation, which occurs in approximately 0.1% to 1.5% of cases [1]. Stapfer et al. classified these perforations into four types based on position and mechanism of injury: Type I (lateral or medial duodenal wall perforation), Type II (peri-Vaterian injury), Type III (distal bile duct injury), and Type IV (retroperitoneal air without obvious perforation) [2]. In our case series of four patients, the clinical presentations, management strategies, and outcomes varied significantly based on the type and severity of the perforation, as well as the patient's comorbid conditions.

The onset of symptoms after ERCP ranged from 12 to 40 hours, consistent with the delayed nature of retroperitoneal perforations (Stapfer Type II) compared to the more immediate presentation of free intraperitoneal air in Type I injuries. In Case 1, the patient presented with retroperitoneal air and mild pancreatitis without overt signs of peritonitis, allowing for initial conservative management. In contrast, Case 2 demonstrated signs of generalized peritonitis with extensive free intraperitoneal air and limited right perirenal retroperitoneal air. Although the perforation site was not visualized intraoperatively, bile-stained intraperitoneal fluid and widespread contamination supported a diagnosis of Stapfer Type I. This pattern suggests a spontaneously sealed anterior duodenal perforation. The clinical presentation of ERCP-related perforations varies widely, ranging from asymptomatic retroperitoneal air to acute peritonitis [4]. Recent case reports indicate that even type IV perforations can be managed conservatively with careful monitoring [6].

Management of duodenal perforation after ERCP is dictated by the type of perforation and the patient's clinical stability. Type I perforations generally require immediate surgical intervention due to the risk of widespread contamination [4,7]. This was evident in Case 2, where early surgery led to the best recovery among the four patients. Type II perforation, on the other hand, can often be managed conservatively if the patient remains hemodynamically stable [3,7]. Recent evidence suggests that the use of self-expandable metal stents (SEMS) in type II perforation cases significantly reduces retroperitoneal abscess formation and shortens hospital stay compared to conventional surgical intervention [5].

While self-expandable metal stents (SEMS) have been suggested in the literature as a minimally invasive option for Stapfer Type II perforations, this approach was not utilized in our series. At our institution, covered SEMS were not available at the time of treatment, and no prior experience existed with post-ERCP perforation stenting. Moreover, the surgical cases in our series either failed initial conservative management (Cases 1 and 4) or had alternative surgical indications, such as necrotizing cholecystitis (Case 3) or Stapfer Type I perforation (Case 2), where SEMS would not be appropriate. Therefore, SEMS was not a feasible or indicated option in these contexts.

In our series, Case 1 received initial conservative treatment with bowel rest, nasogastric decompression, and intravenous antibiotics. Despite intensive supportive care, the patient's condition progressively worsened, but surgical intervention was declined by the family.

In Case 3, surgical intervention was required not only to address the perforation but also to manage the associated complication -- necrotizing cholecystitis. Notably, in Case 4, the decision to operate was driven by clinical deterioration and signs of sepsis despite initial conservative therapy. Intraoperative findings revealed pancreatic necrosis and retroperitoneal abscess, necessitating aggressive intervention. A feeding jejunostomy was created during the procedure to maintain enteral nutrition, facilitating duodenal healing while bypassing the injured segment. Although this approach has not been fully addressed in the ERCP-specific perforation literature, evidence for early enteral nutrition has been shown to significantly reduce in-hospital mortality, pulmonary complications, and hospital/ICU length of stay in patients with gastrointestinal perforation [8].

Nutritional management was individualized based on the patient’s condition and treatment course. In the early phase of conservative management (Cases 1, 3, and 4), bowel rest was implemented with parenteral nutrition to support metabolic needs. Once clinical stability was achieved, oral or enteral intake was gradually introduced. For surgical cases, postoperative nutritional support varied: Case 2 was managed with parenteral nutrition initially, followed by oral feeding; Cases 3 and 4 received early enteral nutrition via jejunostomy in combination with parenteral supplementation. This multimodal approach aimed to maintain adequate caloric intake, reduce infection risk, and support mucosal recovery.

In surgically managed cases, aggressive drainage was achieved using a modified sump drain configuration, consisting of two fenestrated Pezzer catheters inserted into a Penrose drain and positioned in the periduodenal or retroperitoneal region. This setup provided enhanced drainage capacity and minimized the risk of tube obstruction. While routine irrigation was not applied, warm saline flushing was selectively performed in cases of suspected drain dysfunction or ongoing retroperitoneal inflammation, such as necrotizing pancreatitis. Daily clinical and imaging assessments guided decisions regarding flushing or drain adjustment.

Minimally invasive techniques have also emerged as viable options in the management of select duodenal perforations. Among these, endoscopic purse-string suture is a promising alternative to traditional open surgery for small, localized perforations. This technique enables the immediate closure of the defect without the need for extensive tissue dissection, reducing both recovery time and the risk of postoperative complications. A recent case report demonstrated successful closure of an ERCP-induced duodenal perforation using this method, highlighting its potential role in hemodynamically stable patients with contained leaks [9].

The prognosis after duodenal perforation mostly depends on the timing of intervention and the presence of concurrent complications [1,10]. In our series, early surgical intervention correlated with shorter recovery times, as seen in Case 2. Delayed or declined surgical intervention, compounded by underlying liver cirrhosis and ampullary bleeding in Case 1, contributed to suboptimal outcomes. The timing of surgical intervention is a critical determinant of patient outcomes in ERCP-related perforations. Early intervention has been associated with reduced mortality and shorter hospital stay [11,12]. In contrast, aggressive drainage and enteral feeding via jejunostomy in Cases 3 and 4 significantly improved local control of pancreatic inflammation and promoted duodenal healing, reflecting the benefits of comprehensive management strategies in complex perforation cases.

Our case series underscores the importance of early diagnosis and appropriate management strategies according to the type of perforation [3,10]. Contrast-enhanced CT remains the diagnostic modality of choice, guiding decisions for conservative versus surgical intervention [1]. For peri-Vaterian (Type II) perforations, close clinical monitoring and aggressive drainage are key to optimizing outcomes. Furthermore, the role of early feeding jejunostomy in cases with pancreatic involvement may support better nutritional status and recovery. In our experience, placing a feeding jejunostomy during surgery can provide essential enteral nutrition and facilitate duodenal healing, though this approach remains underrepresented in the literature.

## Conclusions

Duodenal perforation after ERCP is a rare but serious complication whose prognosis depends on the type of perforation, the timing of diagnosis, intervention, and comorbidities. In our series, immediate surgery for Stapfer Type I perforation resulted in excellent recovery, while Type II cases benefited from tailored management, including feeding jejunostomy, aggressive drainage, and close monitoring. Early contrast-enhanced CT was crucial in guiding decision-making. These findings underscore the need for a multidisciplinary, individualized approach to improve outcomes. Given the limited sample size of this case series, future studies should further evaluate the role of enteral feeding strategies and minimally invasive techniques in optimizing care.
